# Environmental surveillance detects circulating vaccine-derived poliovirus type 2 that was undetected by acute flaccid paralysis surveillance in 2021 in Uganda

**DOI:** 10.1007/s00705-023-05759-w

**Published:** 2023-04-15

**Authors:** Phionah Tushabe, Josephine Bwogi, James Peter Eliku, Francis Aine, Molly Birungi, Joseph Gaizi, Lucy Nakabazzi, Theopista Kabaliisa, Irene Turyahabwe, Prossy Namuwulya, Mary Bridget Nanteza, Henry Bukenya, Christopher Kanyesigye, Edson Katushabe, Immaculate Ampeire, Annet Kisakye, Barnabas Bakamutumaho, Charles R. Byabamazima

**Affiliations:** 1 Expanded Programme on Immunization Laboratory, Uganda Virus Research Institute, Entebbe, Uganda; 2 National Water and Sewerage Corporation, Kampala, Uganda; 3 World Health Organization, Uganda Country Office, Kampala, Uganda; 4 Ministry of Health Uganda, Kampala, Uganda; 5 WHO Inter-Country Support Team Office for Eastern and Southern Africa (IST/ESA), Harare, Zimbabwe

## Abstract

The success of the global polio eradication initiative is threatened by the genetic instability of the oral polio vaccine, which can result in the emergence of pathogenic vaccine-derived polioviruses following prolonged replication in the guts of individuals with primary immune deficiencies or in communities with low vaccination coverage. Through environmental surveillance, circulating vaccine-derived poliovirus type 2 was detected in Uganda in the absence of detection by acute flaccid paralysis (AFP) surveillance. This underscores the sensitivity of environmental surveillance and emphasizes its usefulness in supplementing AFP surveillance for poliovirus infections in the race towards global polio eradication.

Poliovirus, a member of the genus *Enterovirus* in the family *Picornaviridae* [1], causes poliomyelitis, a childhood disease that can be debilitating, resulting in permanent paralysis and/or death. The polio eradication initiative has been successful largely through use of the oral polio vaccine (OPV), a live attenuated virus vaccine that induces protective mucosal and humoral immunity against paralytic poliomyelitis. However, the emergence of pathogenic vaccine-derived polioviruses (VDPVs) [2] following prolonged gastrointestinal replication, especially in individuals with primary immune deficiencies or in communities with low population immunity due to poor OPV vaccination coverage, is a concern. Prolonged replication is evidenced by nucleotide sequence divergence in the viral protein 1 (VP1) gene from that of the parental OPV strain of > 1% for poliovirus types 1 and 3 and > 0.6% for type 2 [2].

The standard approach to poliovirus surveillance recommended by the World Health Organization (WHO) is the detection and investigation of cases of acute flaccid paralysis (AFP) [3]. This involves standardized virological analysis of two faecal specimens collected 24–48 hours apart from a patient with a suspected infection. However, less than 1% of poliovirus infections result in paralysis, implying that most infected individuals shed the virus without showing any symptoms [4]. The potential for the virus to circulate undetected by the AFP surveillance system has adverse implications for the polio eradication program. Environmental surveillance (ES), the monitoring of polioviruses in environmental specimens contaminated with human faeces, is more sensitive than AFP surveillance [5, 6] and was started in Uganda in May 2017 at four sites in the populous districts of Kampala and Wakiso in central Uganda. The sites included the Kitooro sewage treatment plant (STP) in Wakiso district, Bugolobi STP, Lubigi STP, and the Ministry of Internal affairs sewer inspection chamber, all in Kampala district. These have since increased to 12 (2020, 2 sites; 2021, 2 sites; and 2022, 4 sites) across the country, with one site each in the urban regions of the high-risk districts of Arua (Prison cell STP), Kabarole (Kisenyi sewage ponds), Gulu (Laroo STP), and Moroto (Lia River-Natumukasikou Bridge) and two sites each in Mbarara (Kizungu sewage lagoon and Kakoba sewage lagoon) and Mbale (Doko STP and Namatala STP) (Fig. 1). The above-mentioned districts report influxes of under-vaccinated people from neighboring countries and hence are susceptible to poliovirus importations. We report two circulating vaccine-derived poliovirus type 2 (cVDPV2)  sequences detected in Lubigi STP, five months apart.

**Fig. 1:**
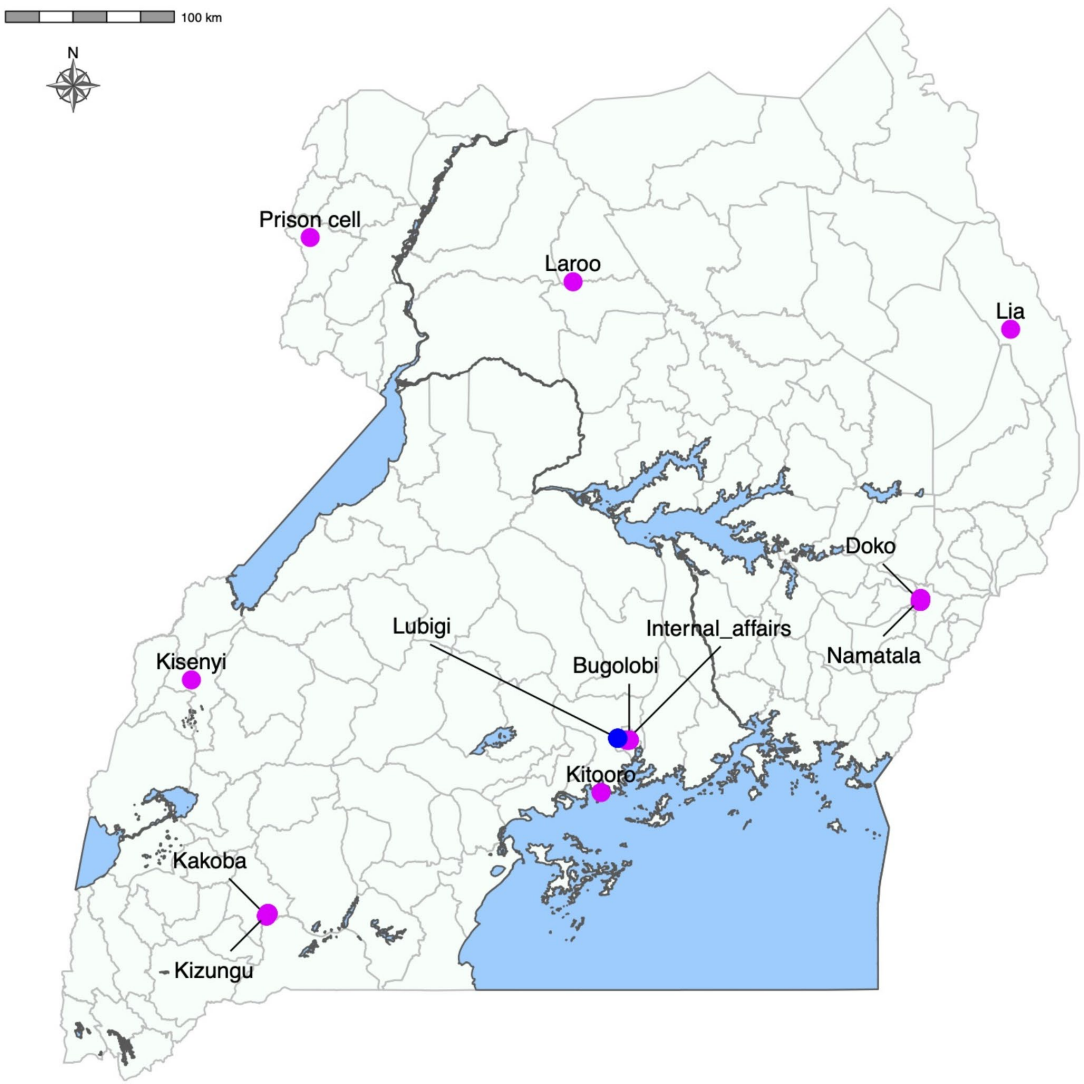
A map of Uganda showing the environmental surveillance sites. Kitooro, Kitooro Sewage Treatment Plant (STP); Bugolobi, Bugolobi STP; Lubigi, Lubigi STP; Internal_affairs, Ministry of Internal affairs sewer inspection chamber; Prison cell, Prison cell STP; Kisenyi, Kisenyi sewage ponds; Laroo, Laroo STP; Lia, Lia River-Natumukasikou Bridge; Kizungu, Kizungu sewage lagoon; Kakoba, Kakoba sewage lagoon; Doko, Doko STP; Namatala, Namatala STP. Lubigi STP, where the cVDPV2s were detected, is indicated in blue.

Grab sewage samples collected at specific intervals and times [7] were shipped under cold chain conditions to the Expanded Programme on Immunization laboratory at the Uganda Virus Research Institute. These were concentrated overnight using the two-phase separation method [8], and the concentrates were treated with chloroform. From the treated concentrate, 0.5 µL was inoculated onto and cultured in the cell lines L20B (a mouse cell line engineered to express the poliovirus selective receptor) and RD (cells derived from human rhabdomyosarcoma) [9] for up to 10 days with a blind passage after 5 days. Positive cultures in the L20B cell line were tested using the intratypic differentiation assay [10], and thereafter, the poliovirus type 2 isolates were spotted onto Flinders Technology Associates (FTA) cards (Whatman, Life Sciences) and shipped to the National Institute for Communicable Diseases (NICD), Johannesburg, South Africa, for sequencing of the VP1 gene. At NICD, RNA was extracted using a QIAamp Viral RNA Mini Kit as described previously [11], and thereafter, the VP1 gene was amplified using specific primers [12]. The 903-nt sequences were determined bidirectionally using a BigDye Terminator Ready Reaction Kit (Applied Biosystems) and a 3500xL Genetic Analyzer (Applied Biosystems). Initial analyses to determine the 903-nt window and genetic relatedness were performed using Sequencher version 5.4.6 (Gene Codes Corporation). Analysis and comparison of these sequences with the Sabin 2 reference sequence (AY082679.1) were also performed using Geneious Prime version 2022.0.2 (Biomatters Ltd), and substitutions in the VP1 gene were seen as nucleotide differences in the sequence alignments.

The two sequences were determined to be circulating vaccine-derived poliovirus type 2 (cVDPV2) and were obtained from samples collected in June (sequence with accession number OP407914) and November (sequence with accession OP407913) 2021. These were from Lubigi, a site that serves the Kampala city suburbs of Kamwokya, Bukoto, Wandegeya, Bwaise, and Mulago (where the country’s National Referral Hospital is housed). Both sequences had the known attenuating mutation I143T in VP1 along with seven other non-synonymous substitutions with a hitherto undefined role in pathogenesis: A9V, K15R, P21L, S23P, N25D, R103K, and S222L. In addition, each sequence had one other non-synonymous substitution: I2T (accession number OP407914) and N171D (accession number OP407913). The sequence OP407914 was genetically related to cVDPV2 sequences that had been detected in 2020 in Sudan, nucleotide sequences that had 1.1–2.8% divergence from the Sabin OPV strain [13]. Sequence OP407914 had 36 nucleotide differences, while sequence OP407913 had 42 nucleotide differences when compared to the Sabin 2 reference sequence, representing 4.0% and 4.7% nucleotide sequence divergence, respectively, suggesting that these viruses could have been circulating uninterrupted for close to 4 years, when assuming an estimated evolutionary rate of 1.03 × 10^− 2^ substitutions/site/year in the P1/capsid region [14].

The World Health Organization conducted a global synchronized switch from trivalent oral polio vaccine (tOPV containing Sabin strains 1, 2, and 3) to bivalent oral polio vaccine (bOPV containing Sabin strains 1 and 3) and the trivalent inactivated polio vaccine (IPV) in April–May 2016. It was anticipated that, with high vaccination coverage, the ‘switch’ would result in improved population serologic and mucosal immunity to types 1 and 3, and with the expected decline in the type 2 population mucosal immunity, protection from paralysis following infection with poliovirus type 2 would be achieved by a single IPV dose [15]. In Uganda, the ‘switch’ was conducted in April 2016, and since then, the WHO estimates of IPV and bOPV3 immunization coverage have consistently been above 80% (Fig. 2) except in 2016 and 2017 [16]. The IPV and bOPV3 coverage for Kampala district, where the Lubigi site is located, has also been consistently above 80%, except in 2020 (Fig. 2). Following confirmation of the first cVDPV2 in June, the collection schedule for Lubigi site was changed to twice a month for the subsequent 11 months to monitor shedding. Hence, a total of 22 samples were tested during this period. Routine AFP surveillance did not identify any cVDPV2 during this period. In addition, a survey of 237 healthy asymptomatic children under the age of 5, the majority of whom were born after the ‘switch’ and hence had received only one IPV dose, was conducted in September 2021, and this did not identify any AFP cases in the site’s catchment areas (unpublished data). This, as well as the absence of the virus in subsequent ES collections, underscores these coverage data, which reflect a high population immunity and pre-empt one to conclude that this was an importation with limited subsequent transmission. However, the cVDPV2 sequences were detected five months apart, which is an indication of sustained shedding. Also, the increase in the number of nucleotide differences between the two sequences indicates continued accumulation of substitutions during circulation. This combination of high genetic diversity and sustained shedding from the population is suggestive of undetected silent transmission [17].

**Fig. 2:**
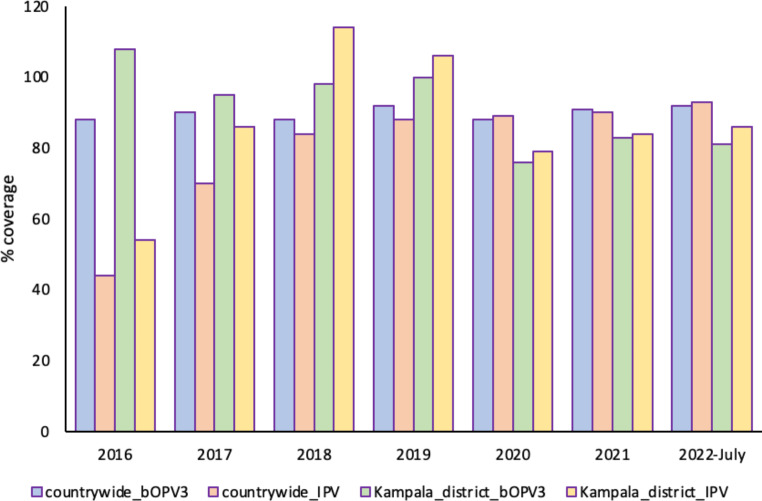
bOPV3 and IPV coverages from 2016 to July 2022 for both the country and Kampala district

Globally, there has been an increase in the cVDPVs reported (Fig. 3) [18], with these exceeding the wild polio types [19]. Of these, cVDPV2 constitutes 95% of all cVDPVs reported, with the bulk of affected countries being in Africa [13]. This is mainly due to the fact that African countries have a comparatively lower routine vaccination coverage than the rest of the world [20].

**Fig. 3:**
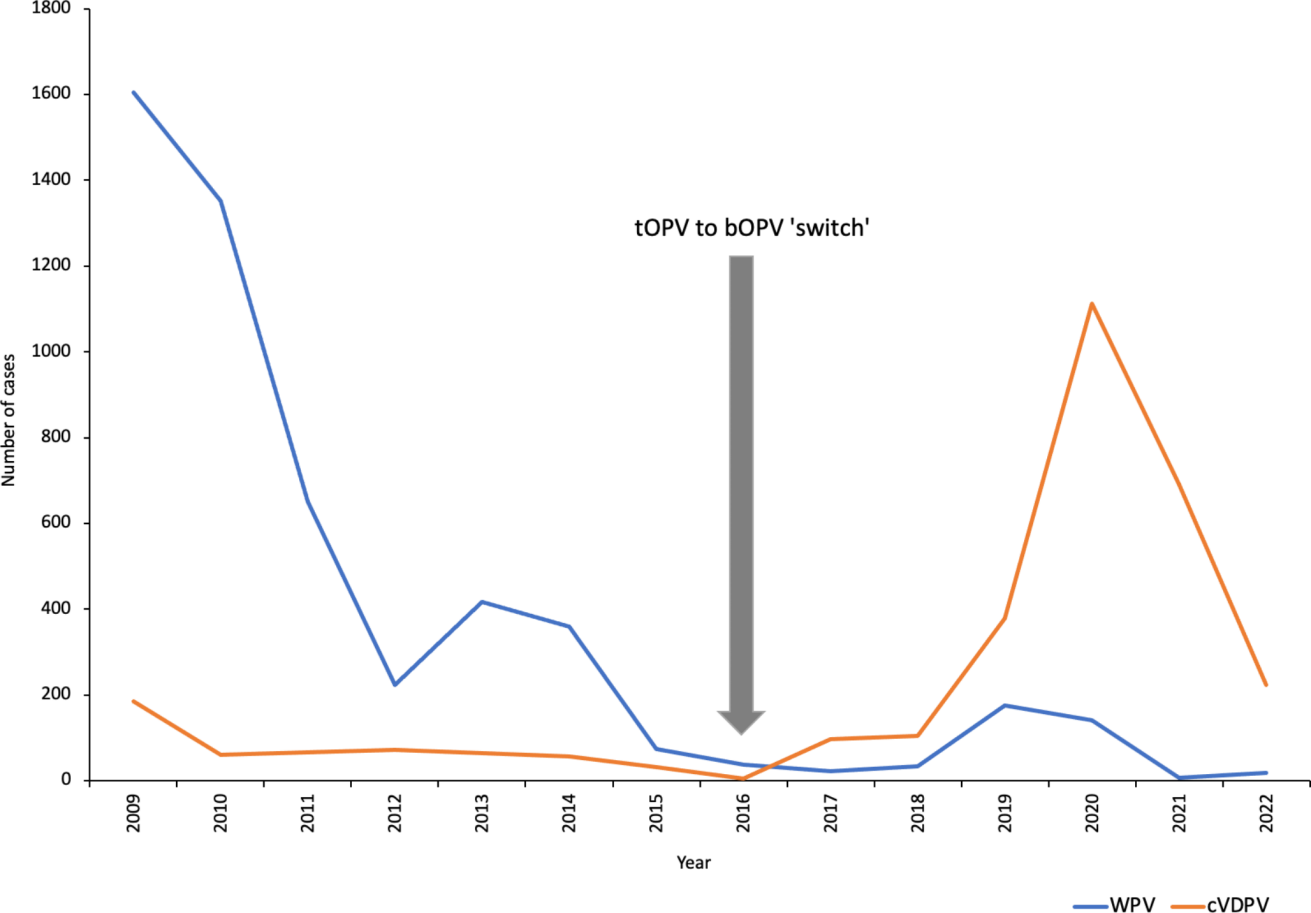
Number of cases of wild poliovirus and circulating vaccine-derived poliovirus detected globally before and after the ‘switch’. The data were obtained from WHO [18]. WPV, wild poliovirus; cVDPV, circulating vaccine-derived poliovirus; tOPV, trivalent oral polio vaccine; bOPV, bivalent oral polio vaccine

The increasing spread and continued evolution of cVDPVs in countries previously declared polio-free is a concern, raising the question of whether the risk mitigation strategies implemented prior to the ‘switch’ were sufficiently effective. Detection of polioviruses (cVDPVs) in environmental specimens only is a growing trend and has recently been reported in Ghana [21] and the United Kingdom [22]. This trend is due to a significant decrease in the sensitivity of AFP surveillance in areas with low prevalence of poliovirus infections as well as in highly immune populations in which polioviruses may circulate without causing paralysis [23]. In addition, metagenomics sequencing of environmental specimens has shown more sensitivity compared to clinical surveillance for other pathogenic viruses such as SARS-CoV-2 and is fast becoming an early-warning tool for impeding outbreaks [24]. With global poliovirus eradication getting closer, routine vaccination coverage – and the resulting population immunity – will need to be maintained at an adequately high level to avert the risk of outbreaks from local evolution and importations. As global poliovirus infections continue to decrease in response to intensified vaccination, enhanced surveillance using environmental specimens will become increasingly critical for timely detection of any circulating polioviruses, especially in countries, like Uganda, with a high rate of influx of immigrants.

## Data Availability

The VP1 sequences described have been deposited in the GenBank databank with the accession numbers OP407913 and OP407914.
